# Distinctive Characteristics of the Female

**Published:** 1847-03

**Authors:** 


### DISTINCTIVE CHARACTERISTICS OF THE FEMALE.

We thank our friend Prof. Meigs, of the Jefferson Medical School, for a copy of his lecture on a subject which he has studied with so much care and interest. It was delivered about the middle of his course, and published at the request of his class. Its object is to show that those who would comprehend the nature and treatment of the diseases of woman, must first make her peculiarities of physiology a special study; and to aid in this, the learned professor, in his own peculiar manner, has presented many of her more striking characteristics, corporeal, intellectual and moral. After speaking of some of the social influences of man and woman, he breaks forth in the following quaint and animated style:

"Now, are these high considerations? and yet from what lowness do they spring! Yea, even from the germiferous tissue of the female. It is from her stroma that issues the generic as well as the genetic force. What a wondrous law! what a wondrous power is that which maintains each genus and species pure and unalloyed as when it issued from the Creator's hand! So great, so powerful, that each of them is set, as it were, within a magic ring, out of whose charmed round it can never stray; so that no wild and horrid passion, no brutal lust, no insane desire can break, much less change or abrogate the law that set forth the primordial models, 'each after his kind,' of the species of the globe. For notwithstanding the countless myriads of generations that from the remotest ages have reproduced individuals more numerous than the sands of the shore or the stars in the firmament, each blade of grass, still obedient to its generic law, still imitates exactly its primitive pattern; and every elephant or worm—every eagle that soars to the sun, or sparrow that chirps in the hedge—every man and every woman go steadily, like the current of a river, down time's flowing stream, ever ending, ever beginning, ever changing, yet immutably the same! I repeat it, the generic power is launched from the ovarian stroma. That is the *sole animal concrete* that is capable of producing yelk matter. Yelk matter is germinal or generic matter—I should rather say, reproductive matter. The male tissues are nowhere endowed with the power of this yelk production, and the sole elaboration of the stroma of ovaries is germ elaboration.

"Let me pronounce three words in your ear: *Stroma is sex.* In pronouncing these words, then, I wish you to appreciate the importance of that tissue—it was for the elaboration of germs that sex was appointed to the female, the human Zygozoaire. See, then, in this unobvious, apparently vile lump of animal texture, in the inner court of the temple of the body, the ark that contains the law, which keeps the genera unmixed, from age to age. How *can* you study this subject sufficiently?"

We are tempted to extract his closing paragraph, in the hope that it may inspire some of our young readers, while many of the older will no doubt find themselves in full sympathy with its ardent and amiable author:

"I do not believe in a physician who knows only calomel and rhubarb. I would have you fill your souls with knowledge; I would have you bathe in it as in an ocean. Were I young again, and could I appreciate, as I now in some degree begin to do, the beauties of learning, I could not cast away as I have done a half century of time; but I would grow pale by the reflection of the midnight lamp, and I would never be satiated until my soul were satisfied with the fullness of knowledge. For what are we in the general but erring and curious inquirers? and does not the most highly cultivated intelligence to be found among men leave them at last, even the most gifted among them, blind, groping, feeble worms of the dust? What should be our motto and our cry, from the lowness of the human nature in which we lie groveling? *Excelsior! Excelsior!*"

